# On ‘modified human agents’: John Lilly and the paranoid style in American neuroscience

**DOI:** 10.1177/0952695119872094

**Published:** 2019-10-09

**Authors:** Charlie Williams

**Affiliations:** Queen Mary, University of London, UK

**Keywords:** brainwashing, Cold War, counterculture, history of psychology, paranoid style

## Abstract

The personal papers of the neurophysiologist John C. Lilly at Stanford University hold a classified paper he wrote in the late 1950s on the behavioural modification and control of ‘human agents’. The paper provides an unnerving prognosis of the future application of Lilly’s research, then being carried out at the National Institute of Mental Health. Lilly claimed that the use of sensory isolation, electrostimulation of the brain, and the recording and mapping of brain activity could be used to gain ‘push-button’ control over motivation and behaviour. This research, wrote Lilly, could eventually lead to ‘master-slave controls directly of one brain over another’. The paper is an explicit example of Lilly’s preparedness to align his research towards Cold War military aims. It is not, however, the research for which Lilly is best known. During the 1960s and 1970s, Lilly developed cult status as a far-out guru of consciousness exploration, promoting the use of psychedelics and sensory isolation tanks. Lilly argued that, rather than being used as tools of brainwashing, these techniques could be employed by the individual to regain control of their own mind and retain a sense of agency over their thoughts and actions. This article examines the scientific, intellectual, and cultural relationship between the sciences of brainwashing and psychedelic mind alteration. Through an analysis of Lilly’s autobiographical writings, I also show how paranoid ideas about brainwashing and mind control provide an important lens for understanding the trajectory of Lilly’s research.

In 1952, the neurophysiologist John C. Lilly accepted a position as head of the Section of Cortical Integration at the National Institute of Mental Health (NIMH) in Bethesda, Maryland. His appointment was based on an ambitious ‘Proposal for a Research Program on the Relations Between the Activities of the Brain, Body and Mind’. Combining communications theory, neurophysiology, and psychoanalysis, his aim was to use new brain-mapping techniques to physically locate and manipulate behavioural correlates in the brains of monkeys, cats, humans, and later dolphins.^1^

In the 1950s, Lilly’s claims to be able to manipulate behaviour also piqued the interest of a number of US military and intelligence officials interested in how new technologies in the neurosciences could be employed to control human and animal minds. In an unpublished paper from the 1950s, Lilly described how his experiments in isolation and neuroelectric stimulation of the brain could be used to modify and control the minds of enemy operatives. Lilly envisioned a not-distant future in which his techniques could be employed to obtain ‘push-button control over the totality of motivation and of consciousness’, leading to ‘master-slave controls directly of one brain over another’.^2^

As the literary scholar Timothy Melley has argued, visions of brainwashing depicting the abuse of the human sciences at the hands of covert institutions were a ‘quintessential fantasy’ of the Cold War (Melley, 2011: 21). The term *brainwashing* first appeared in the early 1950s to describe indoctrination and interrogation techniques used in China and the Soviet Union, but as recent scholarship on the subject has shown, it soon came to represent diverse means of social and behavioural control (Carruthers, 2009; Dunne, 2013; Melley, 2011, 2012). Indeed, Lilly’s work in the 1950s influenced and responded to what historians have called the ‘Cold War brainwashing scare’, and in turn inspired films and literature in which brainwashing was a central plot device (Dearden, 1963; Seed, 2004: 210–15).

Today, however, Lilly is remembered not as an insidious 1950s brainwashing experimenter, but instead as a guru of the counterculture, the human potential movement, and the 1960s psychedelic scene. In the late 1950s, he quit the NIMH to pursue a series of esoteric practices involving human–dolphin communication, psychedelic drugs, and extraterrestrial communication. As the historian Graham Burnett puts it, Lilly went from an ‘Eisenhower-era, pocket-protector-wearing, right stuff engineer’ to a ‘guru-sage of countercultural enlightenment’ (Burnett, 2016). The outcome of Lilly’s work at the NIMH was not, as Lilly had proposed, a detailed translation of brain activity to consciousness and behaviour, but instead a manuscript entitled ‘Programming and Metaprogramming in the Human Biocomputer’. The book was a training manual for the exploration of consciousness, describing how using isolation tanks, psychedelic drugs, and meditation techniques, one could learn to program and reprogram one’s brain.

Lilly is remembered today as the pioneer of flotation therapy, a popular well-being practice in which users float supine on a shallow pool of Epsom salts. Such tanks have been described by some scholars as a 21st-century version of what Michel Foucault called ‘technologies of the self’, techniques and practices that allow individuals to act upon their bodies and minds ‘so as to transform themselves in order to attain a certain state of happiness, purity, wisdom, perfection, or immortality’ (Foucault, 1988: 18).

The aim of this article is to examine Lilly’s shifting career within discourses about brainwashing and mind control, and to show how his research spoke to a series of debates and concerns about the governance of mind in the Cold War. Lilly’s scientific research, it is argued, was caught between two divergent scientific fantasies, one in which his techniques could be used for master–slave control of one mind over another, and another in which his techniques could be used to liberate the mind, renewing a sense of agency that was felt to be either lost or under threat. The first sections will examine Lilly’s brain-mapping project, and the technologies developed that Lilly claimed could be used to intervene upon and manipulate the mind. The article then explores the processes by which Lilly reverse-engineered his techniques as technologies of self. With focus on the isolation tank, I demonstrate how Lilly and his colleague Jay Shurley modified the power dynamics of the isolation experiment, such that control of the experience was placed in the hands of the subject rather than the observer.

This article thus tells a story of reappropriation by Lilly, who took techniques of mind control and reimagined how they could be used in the service of the self rather than against it. In the final section, I consider how this ‘battle for the mind’ plays out in Lilly’s semi-autobiographical work *The Scientist: A Novel Autobiography*. The book, which ran through three editions (1978, 1988, 1997), presents Lilly’s life within a complex exploration of wider belief systems developed inside the isolation tank. Drawing on themes and styles from postwar science fiction and conspiracy novels, *The Scientist* depicts myriad concerns about agency and Lilly’s personal struggle for his own mind. To paraphrase Richard Hofstadter’s famous essay on ‘The Paranoid Style’ (1964), John Lilly is presented in this text as a ‘double sufferer’, afflicted not only by the real world, but by his fantasies as well.

A recent body of scholarship has sought to revisit Lilly’s scientific contribution and the advanced cybernetic and communication theories he employed (Burnett, 2016; Clarke, 2014; Langlitz, 2006; Shiga, 2013). In part, this revived interest can be attributed to a wider scholarly focus on the relationship between science and the counterculture, or what the editors of a recent volume have called ‘groovy science’. David Kaiser and W. Patrick McCray’s *Groovy Science* (2016) challenges Theodore Roszak’s (1969) claim that the 1960s counterculture was primarily antiscientific. Their book examines how, rather than eschewing science altogether, the counterculture embraced certain models of science and scientific practitioners. Graham Burnett’s opening chapter to this volume posits Lilly as the archetypal ‘groovy scientist’, focusing on his enthusiastic uptake of cetacean research, which started life bound up within the military-academic complex, but drifted into more esoteric practices of spiritualism, psychedelics, and consciousness exploration (Burnett, 2016). This paper aims to zero in on Lilly’s research on mind control and consciousness expansion, and situate it within wider discourses about the Cold War ‘battle for the mind’.

At one level, this paper can be read as a very personal account of an unusual scientist. But it would be short-sighted to view the scientific ideas of John Lilly as entirely idiosyncratic, or as preoccupations that were unique to this figure alone. As a historical actor, Lilly offers a model for examining wider concerns about individual agency in Cold War America, emphasising the relationship between brainwashing science and psychedelic culture (see also Holmes, 2017a). Lilly joined the NIMH at the height of the ‘Cold War brainwashing scare’, a period characterised by widespread concerns about the threat posed by the human sciences and new technologies to control human minds. Whilst Lilly’s techniques of brain mapping, electrostimulation and sensory isolation responded to and helped stoke fears of brainwashing in the 1950s and 1960s, he also engaged in and helped develop practices of what he called ‘self-metaprogramming’, using psychedelic drugs and isolation tanks to gain control and mastery over one’s own mind. This paper illustrates the symmetry between these two visions of mind alteration, exploring how they incorporated shared scientific and material cultures whilst speaking to wider cultural concerns about agency in the Cold War ‘battle for the mind’.

## Mapping minds with the bavatron

John Lilly joined the NIMH in 1952. Aged 36, he had already built a promising career in research and was steadily working his way through the US scientific establishment. He studied science at Caltech before training as a doctor at Dartmouth medical school. Hoping to pursue a career in research rather than as a clinician, he transferred to a course at the University of Pennsylvania, where he graduated with a medical degree in 1942. During the Second World War, Lilly took up a position at the University of Pennsylvania on behalf of the US Air Force, where he carried out research on the physiological effects of high altitude (Jeffrey and Lilly, 1990; Lilly, 1978).

Lilly stayed on at the University of Pennsylvania after the war, where he took up a position with the E. R. Johnson Foundation. Between 1946 and 1953, he developed a technique for safely inserting electrodes into the brains of unanaesthetised cats and macaque monkeys. At Pennsylvania, he used this technique to carry out several experiments involving the implantation of 25 electrodes into the cerebral cortex of monkeys. Low-level electric currents produced in the monkey’s brain were then run through an amplifier that could drive 25 analogue light sources, each emitting varying light intensity in proportion to the signal data from the electrodes. This early brain-mapping device, which Lilly called the ‘bavatron’, provided a real-time image of the electrical activity of the brain, which could be recorded using a motion picture camera or represented using an electro-iconogram (Lilly, 1950; Lilly *et al.*, 1955).

In the early 1950s, Lilly began drafting a proposal to carry out a long-term, large-scale research project based on this method. Concerned that the University of Pennsylvania would be unable to support such a project, he sent an early draft of the proposal to the Rockefeller Foundation’s director of medical sciences, Robert Morison. Morison suggested that Lilly apply for a position with the newly formed NIMH under the directorship of Seymour Kety.^3^

The thrust of Lilly’s proposal was to expand the bavatron and the 25-electrode technique dramatically to provide more detailed neuroelectric maps of brain activity. ‘I feel that we are in the position of astronomers who had first [sic] looked through the first crude telescope’, Lilly wrote in a letter to Morison, ‘the 25 channels have given us a first look.…Now we need more channels’.^4^ Such an expansion relied on refinements in method and techniques to allow the safe implantation of what Lilly hoped would eventually be 1000 electrodes. Lilly hoped that this level of detail could be used to correlate neural data with internal and external behaviour, suggesting applications in neuroscience and psychiatry.

In April 1952, the NIMH’s director Seymour Kety suggested that Lilly should apply for the post of chief of the Section of Cortical Integration, promising a generous salary and an initial research budget of approximately $50,000 a year, as well as ‘fairly unlimited facilities and reciprocal stimulation and facilitation among a large number of brilliant scientists’.^5^ Lilly responded with a far more detailed outline of the space and facilities he required, closer to the tune of $100,000 a year over ten years.^6^

## The ‘Cold War brainwashing scare’

Lilly’s appointment to the NIMH came shortly after a new term – *brainwashing* – had been introduced into the English language. The term was first used publicly by journalist Edward Hunter in an article for the *Miami News* (1950).^7^ In this article and in later works, Hunter claimed that by combining Pavlovian theory with modern technology, Russian and Chinese psychologists had developed powerful techniques for manipulating minds. Although it resonated with concerns about the growing global influence of communism, the term *brainwashing* would perhaps never have gained traction if it had not been for a series of scandals involving collaboration between American POWs and the Chinese enemy during the Korean War. Most famously, in 1952, Colonel Frank Schwable and 35 other captured US Air Force personnel publicly confessed to committing crimes of germ warfare against North Korea. Other accounts of collaboration at the hands of the Chinese, including the making of public anti-war and anti-McCarthy broadcasts, received widespread attention during the war. Perhaps most controversially, after a long-awaited armistice deal was agreed in 1953, one British and 21 American POWs refused to be repatriated after the war, choosing to relocate to communist China instead. It was widely reported that the soldiers had been exposed to sophisticated techniques of mental coercion based on Pavlovian science, similar to those reported to have been used to extract confessions for Soviet purge trials such as that of Cardinal József Mindszenty in 1949 (Carruthers, 2009).

Scholarship on this period has described the ‘Cold War brainwashing scare’, the ‘brainwashing idea’, and the ‘spectre of brainwashing’, as a central motif in postwar film and literature upon which myriad concerns about agency and influence were projected (for use of these phrases see, respectively, Carruthers, 1998; Reisch, 2012; Taylor, 2004). Whilst such scholarship has often described brainwashing as a ‘cultural fantasy’, the idea of brainwashing nonetheless had real effects, not least within the human sciences. In the early 1950s, building on investigations carried out since the Second World War, the CIA established its notorious MKULTRA programme, which aimed, in the words of its former director Sidney Gottlieb, to ‘investigate whether and how it was possible to modify an individual’s behaviour by covert means’ (Marks, 1978: 57). What the historian Alfred McCoy has called ‘the Manhattan Project of the mind’ was fuelled by a dual sense of hubris about the CIA’s own research and development potential and paranoia about the capabilities of the enemy, enhanced, as it were, by the semi-tangible reports of enemy scientific projects within what Melley has called the ‘covert sphere’ (McCoy, 2006; Melley, 2012).

According to his own memoirs, Lilly had long been interested in questions of behavioural control. It was reportedly his reading of Aldous Huxley’s *Brave New World* in 1934 and its portrayal of the misuse of the human sciences that influenced his decision to major in biology rather than physics as a student (Lilly, 1997: 57). Although Lilly has somewhat successfully cultivated an image of himself as someone who resisted the lure of military and intelligence funding, like many of his peers, his work and career was heavily shaped by the wider forces shaping the human sciences after the war (Lutz, 1997; McCoy, 2006).

## A trip to McGill

In the early 1950s, on Robert Morison’s suggestion, Lilly paid a visit to Donald Hebb at McGill University. Morison was, at the time, managing Rockefeller grants pertaining to the research of Hebb and Ewen Cameron, who were respectively heads of department in psychology and psychiatry. Both Hebb and Cameron’s projects would become well known for their role in the history of brainwashing and mind control research. The details of Lilly’s trip are unclear, but Rockefeller archives reveal that Lilly was able to view classified experiments into human isolation and forged an important relationship with Hebb.^8^ Lilly would go on to incorporate several aspects of the McGill research into his own research projects at the NIMH.

One of the most important findings to come out of McGill in the 1950s was the discovery of pleasure and reward centres in the mammalian brain by James Olds and Peter Milner. Olds, who was one of Hebb’s postdoctoral students, made the discovery when experimenting with low-voltage electrostimulation on the brains of rats. In one experiment, it was observed that, when stimulated in a certain subcortical area, the rat would return to the location in which the stimulation took place, as if seeking out additional stimulation. Further experiments identified a number of apparent ‘pleasure’ centres. Some of these were so internally rewarding that when a rat was given the means to control stimulation by pushing a lever, it would go on self-stimulating indefinitely, irrespective of basic needs such as rest, hunger, thirst, and sex (Dror, 2016). At the NIMH, Lilly carried out various experiments locating similar pleasure and pain centres in the brains of monkeys (Lilly, 1958a). In these experiments, the monkey was restrained in a specially designed chair and provided with a switch that could be used to either turn on or turn off an electrical current passing through electrodes in its brain. Lilly described these regions within a simple cybernetic framework. Internally rewarding regions that caused the monkey to start the electrostimulation were described as ‘start zones’, whereas regions that caused the monkey to stop a particular behavioural reaction were described as ‘stop zones’. ‘These specific physical operational terms [‘start’/‘stop’] are preferred to “positive” and “negative” reinforcement’, wrote Lilly, ‘which tend to interpret the pattern in learning theory terms’ (Lilly, 1958a: 84; see also Lilly, 1978: 89–90).^9^

At the time of Lilly’s visit, Morison was also handling the Rockefeller grant of Ewen Cameron, whose psychic driving experiments, later covertly funded by the CIA, have made him one of the most notorious and controversial psychiatrists of his generation (Lemov, 2011). Lilly appears to have been impressed by certain aspects of Cameron’s work, in particular the reinforcement of behaviour via repeated signal data through the means of sound recordings on a taped loop. As will be shown in the following section, Cameron’s notion of inserting a dynamic implant into the brain through the repetition of signal data would be incorporated into Lilly’s presentations on the control of human agents.

Of all the research being carried out at McGill, it was perhaps the isolation research, only recently started by Donald Hebb, that would have the greatest influence on Lilly’s life and career. Hebb had recently received an annual research grant of $10,000 from the Canadian Defence Research Board (DRB) to conduct a research project into the effects of perceptual deprivation. The experiment involved placing volunteer research students inside a chamber, specially designed to create an entirely monotonous perceptual environment. In this situation, subjects would hear only the sounds of a white noise generator, their arms would be enclosed within cardboard tubes to restrict movement and tactile perception, and their eyes would be covered by a pair of translucent ski goggles, which allowed in only diffuse white light. Apart from being fed and toileted, subjects would be left in this state for up to three days at a time (Bexton, Heron, and Scott, 1954).

The experiment had been designed in part to test if the subject could be made more suggestible under these conditions. When Lilly visited Hebb in 1952, he would have been one of a small circle of individuals with knowledge of this research, which was only fully declassified by the Canadian DRB in the late 1950s. Hebb had proposed the experiment during a secret meeting at the Ritz Carlton Hotel in Montreal in 1950, in which an international group of medically trained intelligence and defence officials discussed the purported problem of brainwashing and mind control. It was here that Hebb suggested that placing a subject in a situation of total perceptual deprivation might make them ‘susceptible to the implantation of new or different ideas’ (Cooper, 1986: Appendices 21, 6). During the experiments, subjects were given the option to listen to ‘propaganda’ records, which presented evidence in support of paranormal phenomena such as the existence of ghosts. According to Hebb and his students, ‘the effects of propaganda were the only ones that showed signs lasting beyond the experimental period’. Two weeks after the tests, ‘a number of the experimental subjects, unlike the controls, went to the library to borrow books on psychical (*not* psychological) research, mind-reading, and so forth; there were spontaneous reports of being afraid of ghosts, late at night’ (Hebb, 1958: 111); ‘one man even reported that he was trying to use telepathy as an aid to playing poker’ (Heron, 1961: 16).

Although most of Lilly’s time and resources at the NIMH were dedicated to his neuroelectric mapping project, he also devoted two modules of his section and a portion of his research budget to conduct his own sensory isolation research. Using borrowed equipment from Hans Specht at the National Institute of Metabolic disorders, previously used to test the metabolism of swimmers, Lilly devised a unique isolation chamber. The technique involved the total submersion of a subject inside a tank of body-temperature water. As early as 1955, Lilly reported the mind-bending experiences he had after relatively short (compared to the McGill situation) exposures in the tank (Lilly, 1955). In its day, it was known as the most severe sensory-deprivation environment ever developed, and has been the subject of neo-Frankenstein type movie thrillers such as *The Mind Benders* (Dearden, 1963) and *Altered States* (Russell, 1980).

## Building modified human agents

As well as his position as chief of the Section of Cortical integration at the NIMH, Lilly also held a role as surgeon, junior grade, in the Commissioned Officers Corps of the US Public Health Service. Much of what has previously been written about Lilly’s military-oriented work has been lifted directly from his own memoirs, in particular his two autobiographies: *The Scientist* (1978) and *John Lilly, So Far*, co-authored with Francis Jeffrey (1990). In these texts, Lilly claimed to have accepted his quasi-military role because it gave him certain freedoms pertaining to the direction of his research. According to his memoirs (mostly written in the third person), Lilly was approached several times by the CIA and military intelligence services, ‘hoping to get the inside track on his research, hoping to draw him into their game’ (Jeffrey and Lilly, 1990: 91). After carrying out some briefings for military and intelligence agencies, he claimed to have become ‘painfully aware that his research could be used to create powerful brainwashing and mind-control techniques’ (ibid.: 93). This, he claimed, was a key factor behind his decision to leave the NIMH in the late 1950s (Lilly, 1997: 87–97; Jeffrey and Lilly, 1990: 90–4).

To what extent Lilly embraced or resisted military and intelligence interest in his work may never be entirely clear (Burnett, 2012, 2016). However, there are two documents in his personal archive that have hitherto been unexplored by recent Lilly scholars, both of which suggest Lilly’s willingness to align his work with militaristic aims. The first paper is entitled ‘Motivation Control with Electrical Brain Stimulation of Non-Human Species: Communication With Individuals and Use as Agents (Applied Research on Military and Extra-Terrestrial Implications)’. Although it is undated, it is similar in content to one described in both of Lilly’s autobiographies. According to Lilly, he agreed to present a paper on this subject to a group of intelligence personnel at the Pentagon in 1959 (Lilly, 1997: 93–5; Burnett, 2016), on the condition that he would be allowed to talk freely about this work and publish openly. The paper describes how electrostimulation of the ‘start’/‘stop’ mechanisms Lilly discovered in the brains of monkeys and dolphins could be used to speed up learning and control behaviour through direct punishment and reward. Electrostimulation, claims Lilly, allows ‘“push-button” control of the powerful motivational states of pleasure, sex, fear and pain’.^10^

This paper also reveals Lilly’s burgeoning interest in the dolphin as his primary experimental subject. ‘The dolphin’, writes Lilly, ‘with a brain equal to or greater than man’s in size and complexity, learns, with the central electrically-induced motivations, to perfection in about five minutes or less a task which the monkey (one tenth the brain size of man) usually takes several hours to master’. Lilly stresses the humanitarian and philosophical importance of establishing human–dolphin communication, a sentiment for which he would later become well known (Lilly, 1961). But he also points to the potential military value of dolphins, stating that ‘as covert “military” agents or as surprise aids in naval operations, such creatures would be of inestimable help—If they could be induced to side with us instead of with others’.^11^

The second paper from Lilly’s archive (also undated) is not mentioned in his memoirs. Addressed only to ‘intelligence committee D’, it is entitled ‘Special Considerations of Modified Human Agents as Reconnaissance and Intelligence Devices’.^12^ Rather than focusing on behavioural control of animals, it instead considers how his techniques could be applied to human ‘agents’. Lilly opens the paper by framing the problem of human espionage in cybernetic terms, describing the human brain or ‘agent’ as a ‘collector’, ‘storer’, and ‘readouter’ of ‘informational forms of data’. In communications systems between two or more human beings, Lilly explains, the human mind acts as a complex information processor, which can be programmed in different ways that affect how it collects, stores, and delivers certain types of information. Thus, Lilly proposes that the challenge of behavioural control within an espionage network is not only to extract information or protect it from extraction, but also to control how that data is analysed and interpreted by human agents. Lilly describes three methods that can be used to control the internal programming of human agents and insert or extract information: isolation, electromanipulation of stop/start systems, and the direct insertion of electrical data into the brain.

Technically, isolation is described as the simplest method of modifying the internal processing of human agents. If a person is ‘physically and socially isolated by a group long enough’, Lilly explains, ‘he tends to absorb information from selected signals sent into him “on demand” in a way which can profoundly influence subsequent thinking and behavior’. Under these conditions, he writes, there can be an ‘injection of “outside data” into the “inside generators”, with some re-programming developing’. Lilly describes his own research and the aforementioned studies carried out at McGill University. He also suggests that the process of delivering information into the brain could be automated using techniques akin to Ewen Cameron’s method of inserting ‘psychic implants’.^13^ Repeating emotionally important statements on a tape loop, Lilly claims, could have profound effects on thought and behaviour for several weeks.

The second technique described by Lilly is based on electrostimulation of the brain. Citing his own work, Lilly writes that a ‘technique for covert and relatively safe implantation of electrodes into the human brain has been devised’.^14^ Electrostimulation of specific areas, Lilly claimed, could be used to manipulate the emotional state of the subject, bringing about intense states of fear-panic-terror, pain, nausea, fatigue, intense non-sexual and sexual pleasure, and of trance, sleep, and coma. ‘Combined with isolation with an interrogation team’, Lilly writes, ‘such methods probably cannot be withstood by even the strongest personalities—information probably can be elicited or injected readily in a relatively few hours’. Lilly continues, stating, ‘research on these matters is essential for defensive as well as offensive missions’.

The final technique Lilly presents in the paper is described as ‘Direct Electrical Injection – Elicitation of Information from and into the Brain’. By extrapolating his neuroelectric mapping technique and vastly increasing the number of electrodes, Lilly proposes that one could insert neuroelectric datasets, or ‘programs’, into a recipient human brain. This technique, he argues, could allow ‘future control not only of motivation, but of the detailed instant-to-instant thought and pre-thought processes themselves’. If present theory is correct, Lilly continues, this method would lead to ‘master-slave controls directly of one brain over another in greater or lesser degree. The path is a possible one, but the quantities of control achievable are to be determined experimentally’. Lilly concludes this paper stating simply that ‘the ultimate uses of such techniques in the military sphere seem to be obvious’.

The communications scholar John Shiga has argued that Lilly’s techniques for mapping, reconfiguring, and controlling the brain through neuroelectric networks extended what Paul Edwards has called the ‘closed world’ networks of military strategy into the provinces of the mind and brain. In doing so, his work brought cognitive, behavioural, and cultural processes under a framework of systems control (Shiga, 2013). Shiga does not appear to have had access to Lilly’s unpublished papers on the control of human and non-human agents; nonetheless, these papers further demonstrate how Lilly’s research were applied, albeit speculatively, to the pursuit of a brainwashing weapon and methods of engineering the human mind.

## Two lives of the isolation tank

The relationship between Lilly’s isolation tank and brainwashing was first discussed in public by Robert Felix at a hearing in Congress on 14 April 1956. Felix was then director of the NIMH, and his annual report to Congress provided senators with a vivid description of the tank experiments. Felix described how at first the experience could be pleasant, leading to a deep sense of relaxation, but after an hour or two the individual began to discover that their thoughts were ‘going over and over’, until, drawing on Lilly’s language of cybernetics, ‘one is left with a closed circuit’:

Once you have cut these all off and have cut them off long enough so that the person is completely disoriented and disorganized, then if you feed back in information you want this individual to have, and this is the only feed-in he gets, slowly, or sometimes not so slowly, he begins to incorporate this into his thinking and it becomes like actual logical thinking because this is the only feed-in he gets. The problem is that this can happen to any person, some sooner than others. But you can break anybody with this— I don’t care what their background is or how they have been indoctrinated. I am sure you can break anybody with this. (*Labor-Health, Education, and Welfare Appropriations for 1957* , 1956: 596)

The day after the hearing, the *New York Times* published an article entitled ‘Tank Test Linked to Brainwashing’ (1956), detailing much of Felix’s report verbatim. Several other US and Canadian papers picked up on the story, leading to a public relations embarrassment for both Lilly at the NIMH and Donald Hebb at McGill. It was also these reports that inspired the British screenwriter Alexander Kennedy to begin writing a script entitled *The Visiting Scientist*, which would eventually become the 1963 film *The Mind Benders.*

Shortly after Felix’s testimony, Lilly wrote to Donald Hebb and James Olds apologising that this element of his research had been discussed publicly. Hebb replied, advising Lilly to ‘relax, old boy’. So far as the brainwashing reference was concerned, wrote Hebb, the only way this research could have any protective value for the public would be by letting them know about it, continuing, ‘I think this was a good thing to get into the open (though my hands were tied)’.^15^ Publicly, however, Lilly was trying to cultivate an image of isolation, not as a tool of cognitive breakdown and brainwashing, but instead as one that could benefit the human mind. In his first paper on the subject in 1955, Lilly described how prolonged experience inside the isolation tank could lead to ‘a new inner security on a deep and basic level’ (Lilly, 1955: 6). In time, he would argue that if the principles of the isolation tank as a technology of brainwashing were reverse-engineered, it could be used by the individual to gain greater control and autonomy over their own thoughts and actions.

The most significant contributor to the tank project besides Lilly was a Texan psychiatrist named Jay Talmadge Shurley. Shurley joined the NIMH in 1956 in a temporary position as acting chief of the Laboratory of Adult Psychiatric Investigation and chief of the Section on Intrapsychic Dynamics. Shurley was originally appointed as an assistant on Lilly’s project, and was given the technical task of perfecting the many problems with the original mask and thermoregulation equipment. He also trained himself to use the tank and, like Lilly, developed his own unique passion for the experience.

Both Lilly and Shurley were interested in the isolation tank as a tool for exploring the psyche. In early papers, Lilly described his isolation experience in psychoanalytic terms as a regression to the primary process, and argued that his own analytic training under Robert Waelder enabled him to tolerate such disinhibition without anxiety.^16^ As a psychiatrist, Shurley also took an interest in the potential psychotherapeutic uses of the tank. In a diary entry recording his early experiences, Shurley wrote, ‘3 days in the tank could be the equivalent of 3 years of psychoanalysis’. In a later entry, he noted his revelation that this was the most important work he would ever do and that he needed to devote his whole life to it – something that he did for the next fifteen years of his career at the University of Oklahoma.^17^

Following their departure from the NIMH, Lilly’s and Shurley’s careers went in different directions. Arguably their most lasting contribution to isolation research was to develop a set of guidelines for using the tank as a mode of self-analysis and exploration. Lilly and Shurley mapped out the basis of their approach in a 1960 paper, the aim of which, they wrote, was to describe ‘methods of self-observation’ and to establish the physiological and psychological conditions that ‘would give the maximum of information from the subjective sphere’. To do so, they argued, subjects had to familiarise themselves with the tank in three steps, first acting as subject, then as observer – or what they called a ‘safety man’ – and finally as self-observer. Only when the subject had complete control over the experiment were they free to explore their own consciousness with maximum freedom:

For optimal conditions privacy requires the firm assurance to the subject of his control of the secrecy of his individual data indefinitely into the future. Maximum ego freedom and voluntariness of the subject in these experiments can be achieved through such kinds of privacy. It also implies that the subject chooses his own times for beginning, ending, and the total duration of exposure as well as the control of whatever restraints, voluntary inhibitions, and supports are needed. Privacy also implies trust and respect for the ego of the subject on the part of the safety man; i.e. the elimination (or at least attenuation) of any observer encroachment, exploitation, or invasion of each experience and of its data indefinitely (even to the extent of avoiding invited, available, encroachment by the subject). (Lilly and Shurley, 1961: 243)

Lilly and Shurley justified the need for such conditions on epistemological grounds, on the value of eliciting the ‘maximum of information from the subjective sphere’. But their approach can also be read as a subversion of the power dynamics of the experimental situation. In their initial formulation, where sensory deprivation experiments had been described as a method of brainwashing, control had been placed entirely in the hands of the observer. Lilly and Shurley’s approach, on the other hand, radically altered the politics of the experiment, granting total control to the experimental subject instead. This change was crucial in the transformation of the isolation tank’s popular image, from a technology of intervention in the Cold War ‘battle for the mind’ to a democratic technology of human potential and self-actualisation, which resonated with the so called ‘politics of consciousness’ of the 1960s counterculture (Roszak, 1969).

## Self-metaprogramming in the human biocomputer

Lilly’s collaboration with Shurley at the NIMH was short lived. In 1957, Shurley took up a position at the University of Oklahoma, where he pursued isolation experiments in his own purpose-built tank. Although he continued self-experimentation for some time, Shurley’s project also tested the situation on roughly 300 subjects, including potential astronauts, journalists, and skin divers, to test psychological and physiological responses to extreme conditions of isolation (Shurley, 1992). Lilly, on the other hand, established various human–dolphin communication projects at locations in the Virgin Islands, the Bahamas, and California. But he continued his own self experimentation inside the isolation tank for the rest of his life, experimenting with various psychedelic drugs, especially LSD and ketamine.

In 1967, Lilly completed his seminal text *Programming and Metaprogramming in the Human Biocomputer.* It was badly received by his scientific peers and led to the withdrawal of some of his funding. Gradually, however, the text started to gather interest from a different audience. In 1969, following a favourable review in *The Whole Earth Catalog*, 300 mimeographed copies were subsequently sold through the catalogue. The magazine’s editor Stewart Brand later published a larger batch in newsprint (Lilly, 1972: i–iii). Between 1969 and 1972, Lilly took up a residency at the Esalen Institute in Big Sur, California. He also began to fall in with the West Coast ‘hip’ scene, courting friends such as William Burroughs, Alan Ginsberg, and Timothy Leary, as well as Hollywood stars Robin Williams and Jeff Bridges.

*Programming and Metaprogramming in the Human Biocomputer* and his second book, *The Centre of the Cyclone* (1973), remain Lilly’s most widely read works. Both dealt with the exploration of consciousness using sensory isolation, drugs or ‘psychedelic agents’, meditation, and psychoanalysis. Whereas the first was a manual of technique, the second, subtitled *An Autobiography of Inner Space*, provided a more personal description of Lilly’s experiences inside the isolation tank. To his many adherents, Lilly remains today one of the most audacious explorers of consciousness, dedicated to pushing the boundaries of inner experience. ‘In the province of the mind’, his ‘doctrine’ asserted, ‘what one believes to be true is true or becomes true, within certain limits to be found experientially and experimentally. These limits are further beliefs to be transcended’ (Lilly, 1972: xii). In other words, Lilly’s scientific project involved pushing the boundaries of experience and belief to understand to what extent human ‘programming’ was hardwired into the brain, and to what extent the self was amenable to reprogramming and re-modification (Langlitz, 2006):

I would try to go to universes other than our consensus universe, universes I didn’t necessarily believe existed, but which I could imagine. At first this was a test of the hypothesis that what one believes to be true becomes true. Before the trip, I didn’t believe in these universes or spaces, but I defined them as existing. During the LSD trip in the tank I then took on these beliefs as true. After the trip, I then disengaged and looked at what happened as a set of experiences, a set of consequences of the belief. (Lilly, 1973: 48)

Lilly’s attempts to map the boundaries of consciousness also involved turning his own mind into a scientific project, to be remoulded, reprogrammed, and remodified. The psychologist and 1960s psychedelic figurehead Timothy Leary described *Programming and Metaprogramming in the Human Biocomputer* as the ‘*Principia Psychologica* of the Cybernetic Age’, and urged readers to

start thinking of your brain as a biocomputer. Wet Ware. Start thinking of your minds (plural) as your software. You know, sloppy disks that you use to process your thoughts and create images on the screens of your consciousness. This is the basic concept of the cybernetic (information) Age. I consider it the crowning achievement of post-industrial philosophy. (Lilly, 1997: 5)

For Leary, Lilly’s text not only was a manual of phenomenological investigation, but empowered the reader to access the psychedelic ideal of a mutable self that actively rejected socially and culturally conditioned ways of knowing and seeing. Only when we came to understand our intrinsic programming, Lilly wrote, could we free ourselves from ‘the effects of storage of metaprograms which direct our thinking, programs devised by others and fed to us during our learning years’ (Lilly, 1972: xxi). Lilly’s emphasis on self-determination established him as a popular speaker and workshop leader at New Age retreats and institutions like Esalen. This was a community institution of artists, psychologists, and practitioners established in 1962, dedicated to the realisation of what Aldous Huxley called ‘the human potentialities’. Huxley had premised that humans used only a small portion of the conscious and perceptual abilities available to them. Esalen was dedicated to research and teaching that promised to evolve human capabilities and push the boundaries of experience through a combination of New Age meditation, gestalt psychology, Eastern spiritualism, and philosophy. Seminars such as Lilly’s described how psychoactive agents such as psychedelics or sensory deprivation tanks could be used to navigate and unlearn one’s own previous conditioning and open the mind to new possibilities.

Lilly’s method of self-metaprogramming may be described as a modern version of what Michel Foucault calls ‘technologies of the self’. Such technologies, Foucault argues, enable new modes of self-governance by permitting individuals to transform their bodies, minds, beliefs, and behaviour (Foucault, 1988). Today, the floatation tank is one of numerous devices on the market that promise users the ability to manipulate their own brain function in order to improve their psychological lives or mental capabilities. On the one hand, such technologies may be seen as part of several new regimes of self-governance, which enable the self to contort to the demands and pressures of modern life (Brenninkmeijer, 2010; Rose, 1998). Others have argued that self-brain modification can have a more radical political function. The philosopher and historian of science Andrew Pickering, for example, describes Lilly’s isolation tank and other practices of consciousness exploration as a ‘technology of the non-modern self’, as it opened up exploration of inner states of the brain that ‘were not to be ascribed to pure inner causes, but to intersections with the non-self’ (Pickering, 2008, 2010).

In the late 1950s, Lilly had attempted to contain the world of intelligence operatives within a cybernetic, networked discourse, made susceptible to external control. His later texts envisioned similar models of entire societies in which all human beings were connected through networks that were susceptible to external manipulation. As he put it in *Programming and Metaprogramming in the Human Biocomputer*, ‘all human beings, all persons who reach adulthood in the world today are programmed biocomputers. None of us can escape our own nature as programmable entities. Literally, each of us may be our programs, nothing more, nothing less’ (Lilly, 1972: xii). But this text also encoded a set of approaches to understanding and controlling the learned programs of the mind and brain. In other words, a toolkit that enabled readers to transcend their programmed condition and regain a certain mastery or agency over the forces of socialisation, conditioning, and hidden persuasion that shaped them. Lilly couched much of his discussion of the networked mind within an evolutionary theory of the development of information systems, claiming that ‘the cerebral cortex appeared as an expanding new high-level computer controlling the structurally lower levels of the nervous system, the lower built-in programs’. But the human mind, he wrote, also grew the ability to ‘learn to learn’, or what Lilly called ‘self-metaprogramming’ (ibid.).

Melley has argued that Cold War USA was marked by widespread concerns about diminishing agency and autonomy, or ‘agency panic’ (Melley, 2000). Yet if the 1950s had been an era defined by fears of human civilisation reduced to a series of brainwashed automata, Lilly’s research suggested that agency panic also helped create an appetite for technologies or processes of what we might call ‘agency renewal’ amongst emerging generations in the 1950s and 1960s. In the following sections, I explore this relationship between agency panic and agency renewal further, by tracing the role it played within Lilly’s own explorations of the mind. In these writings, self-metaprogramming was presented as a crucial weapon in the 20th-century struggle for the mind, and an antidote to increasingly sophisticated and omnipresent technologies of brainwashing and mind control.

## The scientist and the paranoid style

Lilly left behind numerous autobiographical and semi-autobiographical writings that creatively interwove his personal life, scientific career, altered states experiences, and scientific method into a single narrative. The most revealing of these is his memoir *The Scientist:* *A Novel Autobiography* (1978).^18^ The first edition of *The Scientist* was written during an extended period of experimentation with the tranquiliser ketamine, or what Lilly called ‘Vitamin K’. Lilly had been introduced to ketamine by his friend and fellow researcher Craig Enright in the early 1970s. Yet what Lilly described as a scientific project, exploring alternate realities and his ability to navigate between them, many saw as his downward spiral into addiction. Whilst *The Scientist* certainly reflects the extent of Lilly’s ketamine abuse during this period, as a literary text it also offers a unique insight into his psychic life and the relationship between his scientific research and his own fantasies about the potential of cybernetic systems of control.

In this final section, I will consider *The Scientist* as a literary text that explores myriad concerns about control of the mind, incorporating elements of postwar conspiracy fiction and what Richard Hofstadter called ‘the paranoid style’. As Hofstadter argued in his famous essay of the same name (1964), the paranoid style assumes that ‘a gigantic and yet subtle machinery of influence’ is set in motion to control social and political life. As Melley (2000) has argued, postwar American fiction saw a dramatic intensification of the paranoid style in conspiracy narratives that, rather than resembling small plots of subterfuge and deception, referred instead to the machinations of large organisations, technologies, and systems. Such narratives, Melley points out, often stage contests over the reality of paranoid perceptions versus the consensus narratives accepted by wider society.

Although Lilly is credited as the author, much of *The Scientist* is written in the third person. As in his isolation experiments, this device serves to make Lilly both the observer and the subject of the investigation. The ambiguity over the grammatical person sets the tone for a book that is preoccupied with questions about who, or what, is controlling Lilly’s thoughts and actions. The opening chapter, for example, entitled, ‘A Being Makes a Choice’, posits the biological conception of John Lilly as a moment of self-determination. It describes how a young Lilly made a choice to be male and chose ‘the genetic code that would regulate its form in the future as it grew’ (Lilly, 1978: 19–20). This picture of autonomous self-determination, however, is repeatedly contrasted by Lilly’s belief in insidious and beneficent forms of external control. Raised in a Catholic family, Lilly claimed to readily experience visions of God as a child and believed he was watched over by a guardian angel. Although his scientific training would lead him to eventually reject his early spiritualism, the belief that someone or something is controlling or watching over Lilly appears as a source of both comfort and discomfort in *The Scientist*.

Lilly’s self-analysis in the isolation tank was preceded by his own psychoanalytic sessions under Robert Waelder between 1948 and 1951. In *The Scientist,* Lilly recounts one such session in which he chooses to disassociate from himself and recount his experiences to the analyst as an ‘external being’. Although admitting that this may have been a more tolerable way of penetrating his unconscious, Lilly claims that at this moment in time he prefers not to make any judgements about the reality of the being’s existence. When Waelder suggests afterwards that Lilly should come for seven days instead of five, Lilly accuses Waelder of being frightened by Lilly’s behaviour, and wanting to control and supervise him in detail during a difficult period in analysis (Lilly, 1978: 64–74). His distrust in the psychiatric profession re-emerges in the third edition of the book (1997), when, relating some heavy periods under ketamine, Lilly describes being admitted to a psychiatric hospital in New York. By this time, Lilly was well acquainted with emerging antipsychiatric discourses and, referencing Thomas Szasz’s *Psychiatric Justice* (1965), claimed to have realised that he was ‘in a battle for his freedom’ against the institutional and chemical means of control being imposed upon him (Lilly, 1997: 161–6).^19^

Lilly’s decision to quit his first analytic sessions in the early 1950s was most likely due to his move from Pennsylvania to Maryland rather than an immediate rejection of psychoanalysis. When he first described his experiences inside the isolation tank, they were couched in psychoanalytic terms as a form of ego regression. Yet Lilly came to reject this psychoanalytic language in favour of his own cybernetic framework of programming, arguing that psychoanalytic theory provided inadequate models for understanding the complexity of consciousness when exploring the isolated mind. Indeed, he argued that isolation experiences allowed the mind to connect to domains of reality that were not currently understood by, or did not fit with, contemporary scientific models.

*The Scientist* is punctuated throughout with interactions with what Lilly calls ‘the three beings’. Lilly claims these extraterrestrial beings could influence his life through a process called ‘coincidence control’. Based in the Earth Coincidence Control Office (ECCO), these three beings are described as belonging to an extraterrestrial communications network that Lilly tapped into whilst inside the isolation tank.^20^ In his book, the three beings are described as being able to arrange coincidences in Lilly’s life to help him complete his ‘mission’ on Earth. These coincidences include coordinating the direction of his scientific training, putting him in touch with important colleagues or ‘agents’ on Earth, and at times saving Lilly and his family from near-death experiences. The networks under ECCO’s control resemble both linguistically and conceptually networks of espionage that Lilly had formerly discussed as an employee of the US government. Actors in such networks are often referred to as ‘agents’ in the process of carrying out ‘missions’ and ‘briefs’, and Lilly describes many of the challenges of the 20th century as being rooted in a struggle for influence over the agents in such networks.

As Lilly began to explore his cybernetic spiritualism further, the belief in extraterrestrial controllers appears to have become a source of both comfort and concern. In *The Scientist*, he describes a realisation that modern communications technologies have become more intelligent than humans. During one session under ketamine inside the tank, Lilly begins to envision the future of computers and information networks. As he explains, the human biocomputer consists of organic matter and water, and relies upon the balance of a controlled ecosystem. But around the mid-20th century, he writes, man conceived of solid-state computers, beginning the creation of a new form of intelligence, at first in communications networks and then in computers that could do ‘self-programming as man himself does’. At first, Lilly explains, ‘these networks were ostensibly the servants of humans’. Over time, however, man’s control of what happened in these machines became more and more difficult to maintain, and eventually the solid-state systems began to assume control over the planet and the human species, eventually removing the conditions necessary for organic life (Lilly, 1978: 147–50).

Written in the 1970s, *The Scientist* conveys a worldview that incorporates emerging environmental concerns prominent amongst much of the West Coast counterculture. Lilly claims he saw his experiences inside the isolation tank as a warning that if humans ‘advanced the solid-state entity any further man would eventually become obsolete’. Within this belief system, he came to believe that extraterrestrial solid-state civilisations were trying to manipulate communications networks on Earth, and began to see evidence for this everywhere he went. ‘He finally understood’, he explains, ‘the killing of whales by humans as part of the programming of solid-state intelligences’. The preservation of biological life, he claims, relied upon the reconnection of advanced biocomputers such as those of man and dolphins and organic extraterrestrials. Lilly describes watching a broadcast by former US Attorney General Elliot Richardson during the Watergate scandal, writing,

That man I see on television is a direct agent of the extraterrestrial reality controlling all human life. He is giving a public speech on television to the human species in order to program them into believing that he is not an extraterrestrial agent. In reality, he is controlled by the solid-state life forms of the civilization of another place in our galaxy. It is obvious that what he is saying is to hide his real mission. (Lilly, 1978: 183)

At this moment, the electricity cuts out, which Lilly sees as a sure sign that the solid-state entity is trying to control his own realisation.

Lilly’s voyages into his own consciousness and the vivid writings they produced bring up some fascinating psychic material. As the product of someone whose scientific career had been inspired and enlivened by the potential of the information age to unlock scientific questions about the mind, *The Scientist* is a text that appears to be deeply troubled by it. When we consider how such narratives functioned for Lilly, they can be read at once as far-out speculations of the potential of information communication technologies, and reflections of real-world problems that Lilly was implicated within. As an employee of the American government and military, he had carried out work that explicitly described how minds, thoughts, and actions could be contained within controllable communications networks. Thus, Lilly’s fantasies about total globalised network control can be read as a platform for thinking through the struggle for the mind he believed he was involved in. As Timothy Melley has argued (2012: 70–2), brainwashing theories often avoid structuralist approaches to understanding the mind, by staging a contest between the will of the individual (in this case Lilly) and the will of an exceptionally powerful, rational, and malevolent agent (the brainwasher). Isolation, psychedelics, and self-metaprogramming were promoted as methods of renewing or protecting one’s sense of agency within manifold systems of control and surveillance. To complicate and fragment the idea of self by hacking the mind’s internal programming was seen as one way of making the self both robust and resilient to external manipulation.

## Conclusion

What can this close reading of the scientific journeys and the animate psychic life of John Lilly tell us? On first reading, he might be dismissed as an eccentric, an anomaly who abandoned his mainstream scientific career to pursue a hedonistic course of research in esoteric science. Unusual he may have been, but his scientific and literary work resonated deeply with wider concerns or even cultural fantasies of the Cold War era. This research responded to and fuelled fears of brainwashing and mind control. But it also helped establish a series of technologies and techniques that promoted the potential for self-discovery and agency renewal. For those who practised it, self-metaprogramming was a way of disentangling oneself from other-directed programming and social conditioning. Tracing the trajectory of Lilly’s career demonstrates how the history of brainwashing research and psychedelic technologies of self can be read as intellectually and psychically interdependent, underpinned by widespread concerns about agency in the postwar period.

This article has presented two histories of Lilly in confluence with one another. The more orthodox one deals with his scientific career, tracing his work alongside the intellectual, institutional, and cultural concerns of its time. But it has also taken an approach that engages with Lilly’s journeys as a psychonaut. Through such analysis, we can see that Lilly engaged in elaborate fantasies about the potential of his techniques both as a means of controlling brains, and as technologies of mental liberation that could be used to free the self from the tyranny of programming and social conditioning. Scholars of the Cold War brainwashing scare as well as the psychedelic movement have often implicitly or explicitly argued that brainwashing and consciousness expansion were cultural fantasies of the period. Analysing the career of John Lilly demonstrates the empirical basis of such fantasies and the impact they had upon science, culture, and the psyche of individual researchers.

Today, one of the spheres in which Lilly continues to loom as a prominent figure is the contemporary floatation therapy industry, which has grown almost exponentially in North America during the last five years. Lilly has long been regarded as both the founder and an intellectual guru of floatation. As I discovered in 2016 at an industry conference in Portland, Oregon, his legacy is also a controversial topic. Many within the industry want to shed the esoteric, New Age image associated with Lilly, and situate the practice of floatation within the world of consensus science that Lilly himself eschewed.^21^ A further source of controversy for the floatation industry is its association with interrogation and brainwashing. In the late 1960s and early 1970s, various publications sought to expose the relationship between academic sensory deprivation research and military funding, leading in part to a decline of sensory deprivation research in North America (Raz, 2013; Suedfeld, 1980). As this paper has demonstrated, Lilly played a role in shaping both of these more sensational images of sensory deprivation as a technology that could be used to control minds, but also as one that could be used to make the mind robust and resistant to external control. This idea was by no means new. Isolation has throughout history been a means of bringing about profound changes in self. Lilly and the Cold War psychological arms race of the 1950s leant the practice a technoscientific agenda, fuelling new approaches and understandings of the brain, and of modelling and remodelling the self.

## Notes

This article was adapted from a chapter of my PhD thesis *Battles for the Mind: Brainwashing Altered States and the Politics of the Nervous System (1945-1970)*, completed at Birkbeck in 2018. I would like to thank my anonymous reviewers, my supervisor Professor Daniel Pick, my examiners Professor Susan Carruthers and Dr Edmund Ramsden, and my colleagues in the Hidden Persuaders research group Marcie Holmes, Ian Magor, Katie Joice, and Sarah Marks for their helpful comments on this article. Conversations with Lilly’s biographer Joshua Horwitz, as well as Andreas Killen and Vassili Christodoulou, were also particularly useful in compiling material for this article. I would also like to thank participants at the conference *The Paranoid Style Revisited* at the University of Manchester in June 2018, where a version of this paper was presented.

1. John Lilly, ‘MS: Proposal for a Research Program on the Relationship Between the Activities of the Brain, Body and Mind’, 1952, John Cunningham Lilly (1915–2001) papers, Special Collections and University Archives, Stanford University (hereafter, Lilly Papers), Box 31, Folder 15.

2. Lilly, ‘Special Considerations of Modified Human Agents as Reconnaissance and Intelligence Devices (Committee D, Intelligence and Reconnaissance)’, Lilly Papers, Box 54, Folder 17.

3. Correspondence between Lilly and Morison can be found in Lilly Papers, Box 29, Folder 8.

4. Lilly to Morison, 26 October 1951, Lilly Papers, Box 29, Folder 8.

5. Kety to Lilly, 30 April 1952, Lilly Papers, Box 29, Folder 8.

6. Lilly to Kety, 2 May 1952, Lilly Papers, Box 29, Folder 8. The figure of $100,000 is based on Kety’s response that he expected the project to cost $1 million over ten years: Kety to Lilly, 8 May 1952, Lilly Papers, Box 29, Folder 8.

7. For more on the origins of the term *brainwashing*, see Holmes (2017b).

8. An excerpt from a Robert Morison diary in a Rockefeller file related to Hebb’s grant dated 10 April 1954 reads, ‘John Lilly, who is temporarily at the National Institute of Health, has been sufficiently excited by his visit to Donald Hebb (on RSM’s recommendation) that he has undertaken to repeat the human isolation experiments with a somewhat different technique’. Rockefeller Foundation, ‘Rockefeller Foundation Records, Projects, RG 1.2’, Subseries 427.A: Canada – Medical Sciences, Box 4, Folder 33.

9. Lilly described the ‘start’/‘stop’ model of brain function further: I leave you with one general ‘interdisciplinary’ proposition to question: the initiation and the repetition of all (motor) actions of themselves are either internally rewarding (‘start’ effect) and/or cause the termination of internal punishment (‘stop’ effect). This may sound ‘psychological’, but by ‘internally’ and ‘internal’ I mean ‘within the brain’, and I do mean specific basic neurophysiologic activities yet to be delineated when I talk about ‘reward and punishment’! The ‘start’ and the ‘stop’ patterns…must permeate all of the animal’s and all of the human’s life to assure continuous and constant attention to that which leads to survival of the body, of the species, and even of institutions. (Lilly, 1958a: 720)

10. In his memoirs, Lilly wrote that a company called the Sandia Corporation developed a technique based on his research, which involved steering a mule across a mountain range using a sun compass: ‘The sun compass was hooked to the brain electrodes so that if the mule deviated from his course he was punished, and if he remained on course he was rewarded.…The mule could also be radio controlled from a distance to change his course’ (Lilly, 1978: 96–7).

11. Lilly, ‘Motivation Control With Electrical Brain Stimulation of Non-Human Species: Communication With Individuals and Use as Agents (Applied Research on Military and Extra-Terrestrial Implications)’, Lilly Papers, Box 29, Folder 7.

12. Lilly, ‘Special Considerations of Modified Human Agents as Reconnaissance and Intelligence Devices (Committee D, Intelligence and Reconnaissance)’, Lilly Papers, Box 54, Folder 17. Exactly when and where this paper was delivered is unclear. In his paper on human manipulation, Lilly implies that both papers were presented at the same place or at connected events: ‘In the following discussion I wish to mention a few human cases; a later supplement will mention certain non-human species as possible agents.’ It is likely that this work or similar was presented at the Pentagon meeting discussed in Lilly’s memoirs (Lilly, 1997: 93–5). According to a former intelligence official’s description of this meeting ‘Dr. Lilly stated that the potential of this technique in “brain-washing” or interrogation or in the field of controlling the actions of humans and animals is almost limitless’: memorandum, Jones to Deloach, 2, FBI personal file, as quoted in Burnett (2016). However, an old inventory for Lilly’s archive from 1992 includes the entry ‘Manuscript of Presentation Given to GAP Symposium on Brainwashing Entitled: “Special Considerations of Modified Human Agents as Reconnaissance and Intelligence Devices”: November 1956’, suggesting that Lilly may have delivered this paper at a meeting for the Group for the Advancement of Psychiatry, which we know he attended (Group for the Advancement of Psychiatry, 1956). Yet this is further complicated by the fact that both papers include references dated as late as 1958, suggesting the papers in the archive were presented after 1956.

13. The Cameron reference Lilly cites is Cameron (1950: 2–8).

14. The paper Lilly cites is Lilly (1958b).

15. Hebb to Lilly, 19 May 1956, Lilly Papers, Box 29, Folder 16.

16. This is recorded in a diary entry by Robert Morison dated 10 April 1954: Rockefeller Foundation, ‘Rockefeller Foundation Records, Projects, RG 1.2’, Subseries 427.A: Canada – Medical Sciences, Box 4, Folder 33.

17. Shurley went on to build what he called ‘the Cadillac of isolation tanks’ for roughly $20,000 and, under initial guidance from Lilly, continued their collaborative research, testing the situation on almost 300 subjects. Reports and data from this research can be found in the Jay Shurley Papers at the Robert Bird Library, University of Oklahoma.

18. Editions two (1988) and three (1997) were subtitled *A Metaphysical Autobiography*.

19.
Szasz’s *Psychiatric Justice* (1965) is not mentioned in the first edition of *The Scientist*, but Lilly does describe his psychiatrist as attempting to ‘impose control over my mood and my state of being through chemical means’ (Lilly, 1978: 165).

20. Lilly expanded on his belief that isolation and ketamine could be used to increase the communicative power of the brain in an interview in 1993: The brain, the biocomputer, is a huge transmitter/receiver, and we’re just beginning to see what it is. Have you ever seen anything like a TV show on ketamine? They’re real. The first time I saw that I thought, ‘My God, all we’re doing is increasing the sensitivity of the brain to microwaves’. And the problem with microwaves is that they’re influencing us below our level or awareness all the time. This morning for instance, on ketamine, I went into this place where all these people were interacting and I got involved. When I came back I realized that I had got into a soap opera on TV and was taking part in it as if it was reality!’ (quoted in McClen and Brown, 2013)

21. Clinical trials into the benefits of floatation are now being carried out by Justin Feinstein and a team of researchers at the Laureate Institute of Brain Research, Tulsa, Oklahoma (Oaklander, 2015).
